# Efficacy of surgical antiseptic handrub containing PHMB

**DOI:** 10.1186/1753-6561-5-S6-P270

**Published:** 2011-06-29

**Authors:** E Noguchi, M Yamamoto

**Affiliations:** 1 Biochemical laboratory, Saraya Co. Ltd., Osaka, Japan

## Introduction / objectives

The efficacy and cytotoxicity of PHMB (polyhexamethylene biguanide) -containing surgical hand rub was investigated.

## Methods

Alcohol proportion and bactericidal agents (e.g. CHG (chlorhexidine gluconate), BAC (benzalkonium chloride), PHMB and OCT (Octenidine)) were compared to determine suitability for surgical hand antisepsis. PHMB-containing handrub was then tested in accordance with European Norm (EN). To further evaluate the hand antiseptics or bactericidal agents, their cytotoxicity was investigated with normal human epidermal keratinocyte cells or 3D Human skin model (Vitro-life skin, Gunze).

## Results

The mixture of IPA (isopropanol) and PA (propanol) showed superior efficacy to either IPA or EtOH (ethanol) alone, whereas, for the bactericidal agents, PHMB exhibited the better bactericidal efficacy but lower cytotoxicity. After performing several tests according to EN for hand antiseptic, the PHMB-containing handrub (Alsoft E, Saraya Co., Ltd.) meets EN1500 for hygienic handrub and EN12791 for surgical handrub requirements in relatively short application time 15 seconds and 1 minute, respectively.

## Conclusion

The hand rub containing IPA, PA and PHMB (Alsoft E) exhibited the excellent bactericidal efficacy in relatively short time and lower cytotoxicity, suggesting it suitable for fast-acting surgical hand disinfection.

## Disclosure of interest

None declared.

